# Sympathetic Pathways Target Cholinergic Neurons in the Human Colonic Myenteric Plexus

**DOI:** 10.3389/fnins.2022.863662

**Published:** 2022-03-17

**Authors:** Dominic R. Parker, Lukasz Wiklendt, Adam Humenick, Bao Nan Chen, Tiong Cheng Sia, David A. Wattchow, Phil G. Dinning, Simon J. H. Brookes

**Affiliations:** ^1^Laboratory of Neurogastroenterology, College of Medicine and Public Health, Flinders University, Adelaide, SA, Australia; ^2^Colorectal Surgical Unit, Division of Surgery, Flinders Medical Centre, Bedford Park, SA, Australia

**Keywords:** enteric nervous system, myenteric plexus, sympathetic nervous system, colon, human, gastrointestinal motility

## Abstract

**Background:**

The sympathetic nervous system inhibits human colonic motility largely by effects on enteric neurons. Noradrenergic axons, which branch extensively in the myenteric plexus, are integral to this modulatory role, but whether they contact specific types of enteric neurons is unknown. The purpose of this study was to determine the association of noradrenergic varicosities with types of enteric neurons.

**Methods:**

Human colonic tissue from seven patients was fixed and dissected prior to multi-layer immunohistochemistry for human RNA binding proteins C and D (HuC/D) (pan-neuronal cell body labelling), tyrosine hydroxylase (TH, catecholaminergic labelling), Enkephalin (ENK), choline acetyltransferase (ChAT, cholinergic labelling) and/or nitric oxide synthase (NOS, nitrergic labelling) and imaged using confocal microscopy. TH-immunoreactive varicose nerve endings and myenteric cell bodies were reconstructed as three dimensional digital images. Data was exported to a purpose-built software package which quantified the density of varicosities close to the surface of each myenteric cell body.

**Results:**

TH-immunoreactive varicosities had a greater mean density within 1 μm of the surface of ChAT +/NOS− nerve cell bodies compared with ChAT−/NOS + cell bodies. Similarly, ENK-immunoreactive varicosities also had a greater mean density close to ChAT +/NOS− cell bodies compared with ChAT−/NOS + cells.

**Conclusion:**

A method for quantifying close associations between varicosities and nerve cell bodies was developed. Sympathetic axons in the myenteric plexus preferentially target cholinergic excitatory cells compared to nitrergic neurons (which are largely inhibitory). This connectivity is likely to be involved in inhibitory modulation of human colonic motility by the sympathetic nervous system.

## Introduction

The sympathetic nervous system modulates the functions of the gastrointestinal tract (GIT) (Lomax et al., 2010). In addition to causing vasoconstriction and reduced secretomotor function, sympathetic activity inhibits gut motility. Post-ganglionic sympathetic neurons to the GIT are noradrenergic, with cell bodies in the prevertebral and paravertebral ganglia, which project through the extrinsic mesenteric nerves to enter the intestinal wall (Wood, 1999).

In animal and humans, sympathetic activity inhibits motility by two mechanisms. Sphincteric smooth muscle receives a direct noradrenergic innervation and contracts in response to sympathetic activation, slowing the progression of content through the GIT (Costa and Gabella, 1971; Gillespie and Maxwell, 1971). In contrast, sympathetic axons are generally sparse in non-sphincteric smooth muscle. The myenteric plexus is also a major target of sympathetic axons (Norberg, 1964; Jacobowitz, 1965). Functional studies demonstrated that noradrenergic nerve activity inhibits the release of acetylcholine from enteric nerve terminals (Kewenter, 1965; Jansson and Martinson, 1966; Beani et al., 1969; Hulten, 1969; Jansson et al., 1969; Del Tacca et al., 1970) *via* presynaptic inhibition (Nishi and North, 1973; Hirst and McKirdy, 1974) mediated primarily by alpha 2a adrenergic receptors (Drew, 1978; Stebbing et al., 2001; Scheibner et al., 2002). However, noradrenaline and agonists also cause membrane hyperpolarisation of some myenteric neurons (Galligan and North, 1991) which may contribute to rare inhibitory post-synaptic potentials recorded in myenteric neurons.

The association between noradrenergic terminals (axon varicosities) and intrinsic cells of the myenteric ganglia remains poorly characterised. Using electron microscopy, several studies have reported synaptic contacts between sympathetic endings and myenteric nerve cell bodies (Manber and Gershon, 1979; Gershon and Sherman, 1982, 1987; Llewellyn-Smith et al., 1984).

The specific details of these connections may be important in helping us to understand the causes of functional colonic disorders in humans. For instance, post-operative ileus, the temporary cessation of GIT motility following surgery, has a significant sympathetic component (Behm and Stollman, 2003; Bauer and Boeckxstaens, 2004; Wattchow et al., 2020). Slow transit constipation is a debilitating disorder with a largely unknown aetiology, however, extrinsic sympathetic inputs running in the extrinsic nerves have been implicated (Dinning et al., 2016). Gaining insight into the specific types of myenteric neurons targetted by varicose neurotransmitter released from sympathetic axons is therefore of clinical relevance.

In this study our aim was to determine if noradrenergic (post-ganglionic sympathetic) nerve terminals target specific neurons in the myenteric plexus of the human colon and compare this to enkephalin-immunoreactive axons which arise primarily from enteric ascending interneuronal pathways and thus provide a comparison group from a different source (Brookes et al., 2020; Humenick et al., 2020).

## Materials and Methods

### Colonic Tissue Collection

Specimens from eight patients were collected for this study. Due to high background labelling, one of these was discarded. For the seven specimens analysed, the median age was 66 years (range 37–90), five patients were female, and there were two specimens for each of the ascending, transverse and descending colon, with one specimen from the sigmoid colon.

Human colonic tissue was acquired from the patients undergoing surgical colonic resection for cancer. Ethics approval for this project was obtained from the Southern Adelaide Clinical Human Research Ethics Committee, approval number 207.17. All patients had the process of tissue donation explained to them and signed a written consent form, and all specimens were de-identified. Only non-cancerous regions were examined.

### Colonic Tissue Collection and Preparation

At the time of resection, a 2 cm ring of tissue was cut from the uninvolved healthy margin of the colonic specimen. The colonic tissue was transported in oxygenated Krebs solution (pH; 7.4, NaCl; 118 mM, KCl; 4.8 mM, CaCl_2_; 2.5 mM, MgSO_4_; 1.2 mM, NaHCO_3_; 25 mM, NaH_2_PO_4_; 1.0 mM, glucose; 11 mM, bubbled with 95% O_2_ and 5% CO_2_) from the operating theatre to a laboratory in the same building. The colonic tissue was pinned out in a Sylgard-lined petri dish (Dow Corning, Midland, MI, United States) under a dissecting microscope. Oxygenated Krebs solution was regularly changed. The mucosa, submucosa and some of the serosa were removed by microdissection. The tissue was then stretched maximally in both longitudinal and circular axes and fixed with 4% paraformaldehyde (pH 7.2, 4% paraformaldehyde in 0.1 M phosphate buffer) overnight at 4°C. It was then further fixed in 4% paraformaldehyde on an orbital mixer at room temperature, for a total of 24 h, thus ensuring penetration of the fixative. Subsequently the tissue was further dissected in 1× phosphate buffered saline (PBS) to remove most of the circular muscle and any remaining submucosa and serosa. This produced a wholemount of longitudinal muscle and myenteric plexus, from which a 10 mm × 15 mm specimen was cut from a region between the taenia coli.

### Multiple Layer Immunohistochemistry, Confocal Microscopy, and Antibody Elution

Antisera to human RNA binding proteins C and D (HuC/D), a pan-neuronal marker, tyrosine hydroxylase (TH), an enzyme integral to catecholamine biosynthesis and leu-Enkephalin (ENK), a neuropeptide expressed by some classes of enteric neurons were applied to specimens for 2–4 days at room temperature. The primary and secondary antibodies used are shown in Table 1. The HuC/D antibody was incubated with a biotinylated secondary antibody for a 6-h period, followed by Streptavidin AMCA (aminomethylcoumarin) overnight, producing intense blue immunofluorescence. The tissue preparations were mounted in 100% buffered glycerol on a glass slide and cover-slipped.

**TABLE 1:** First and second layers of immunohistochemistry, primary and secondary antibodies.

	Species	Manufacturer	Ref number	Concentration used	Duration
**First layer, primary antibodies**					
HuC/D	Mouse	Molecular probes	1986151	1:200	3 days
Leu-ENK	Rabbit	ImmunoStar	20066	1:1,000	3 days
TH	Sheep	Millipore	AB1542	1:1,000	3 days
**First layer, secondary antibodies**					
Biotin	Donkey anti-mouse	Jackson	62976	1:100	6 h
Streptavidin AMCA		Jackson	54773	1:100	Overnight (16–18 h)
AF488	Donkey anti-rabbit	Invitrogen	Ref A21206 Lot 1480470	1:1,000	Overnight (16–18 h)
CY5	Donkey anti-sheep	Jackson	87481	1:200	Overnight (16–18 h)
**Second layer, primary antibodies**					
ChAT	Rabbit	Schemann	P3YEB	1:1,000	3 days
NOS	Sheep	Emson	K205	1:5,000	3 days
**Second layer, secondary antibodies**					
CY3	Donkey anti-rabbit	Jackson	711-165-152	1:200	Overnight (16–18 h)
AF488	Donkey anti-sheep	Jackson	713-545-147	1:1,000	Overnight (16–18 h)

*Leu-ENK, Leu-Enkephalin; TH, Tyrosine Hydroxylase; AMCA, aminomethylcoumarin; AF488, Alexa Fluor 488; CY5, cyanine dye 5; ChAT, choline acetyltransferase; NOS, nitric oxide synthase; CY3, cyanine dye 3.*

A confocal microscope (Zeiss LSM880) was used to image 6–7 individual ganglia from each preparation. Ganglia with good quality immunohistochemistry, low background noise, consistent labelling by antisera, with few artefacts and without damage were chosen. Images were captured with 20× magnification and other parameters (exposure times) were kept uniform throughout. Each ganglion was imaged using the Z-stack function with 1 μm “Z-steps” between individual slices, care was taken to include all varicosities associated with the HuC/D cell bodies. This produced a 3-dimensional image of each ganglion.

After visualisation, preparations were then treated with 50 mls of 2-mercaptoethanol/SDS buffer (2-ME/SDS) (Gendusa et al., 2014) in a pre-heated waterbath (56°C) and allowed incubate for 1 h with agitation at 60 rpm. Afterward, the colonic tissue was removed and washed in PBS-Tween (Sigma Aldrich, North Ryde, NSW, Australia) three times for 10 min, before being washed in standard 1% PBS. This eluted the primary and secondary antisera, with the exception of the HuC/D labelling. Due to the high-affinity of the biotin-streptavidin bond, staining for HuC/D was not affected by the 2-ME/SDS solution. Specimens were then re-mounted in buffered glycerol, and viewed and photographed using standard exposures on an epi-fluorescence microscope to check that the TH and ENK immunoreactivity had been effectively eluted.

**FIGURE 1:**
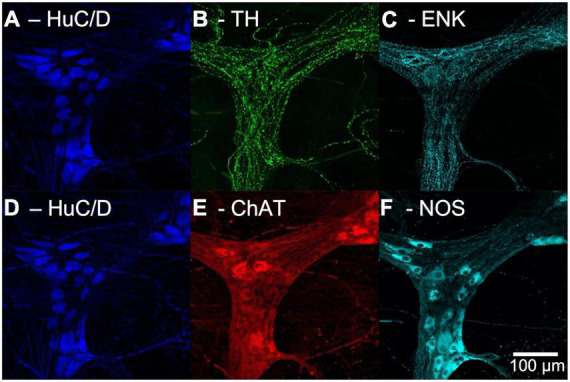
Confocal images of multi-layer immunohistochemistry of a single ganglion in the myenteric plexus of human colon. Panels **(A–C)** show the first layer of immunohistochemistry. **(A)** Human RNA binding proteins C and D (HuC/D), a marker for neuronal cell bodies, shown in blue. **(B)** Tyrosine Hydroxylase (TH) a marker for noradrenergic varicosities, in green. **(C)** Enkephalin (ENK) varicosities, in cyan. Panels **(D–F)** show the second layer of immunohistochemistry. **(D)** HuC/D labelling is retained after elution due to the high affinity Biotin-Streptavidin bond. **(E)** Choline acetyltransferase (ChAT), a marker for cholinergic neurons, shown in red. **(F)** Nitric oxide synthase (NOS), a marker for nitrergic neurons, shown in cyan.

After unmounting, washing with PBS, clearing with 0.5% Triton™ X-100 in PBS overnight and a further wash with PBS, the preparations were immersed in antisera to choline acetyltransferase (ChAT), a marker of cholinergic cell bodies, and nitric oxide synthase (NOS), a marker of nitrergic cell bodies, followed by appropriate secondary antisera (see Table 1). Specimens were remounted in 100% buffered glycerol and the same ganglia were imaged on the confocal microscope. This protocol made it possible to identify whether each HuC/D-labelled nerve cell body was immunoreactive for ChAT and/or NOS. An example of confocal images from a single ganglion in the myenteric plexus put through multilayer immunohistochemistry is shown in Figure 1.

### Imaris 3 Dimensions Reconstruction of Cells and Varicosities

Two ganglia from each preparation were reconstructed in 3D using confocal Z-stack images and were uploaded to the software package Imaris (Bitplane AG, Imaris x64, version 8.4.1). Ganglia were reconstructed individually for HuC/D, TH and ENK immunoreactivity.

**FIGURE 2:**
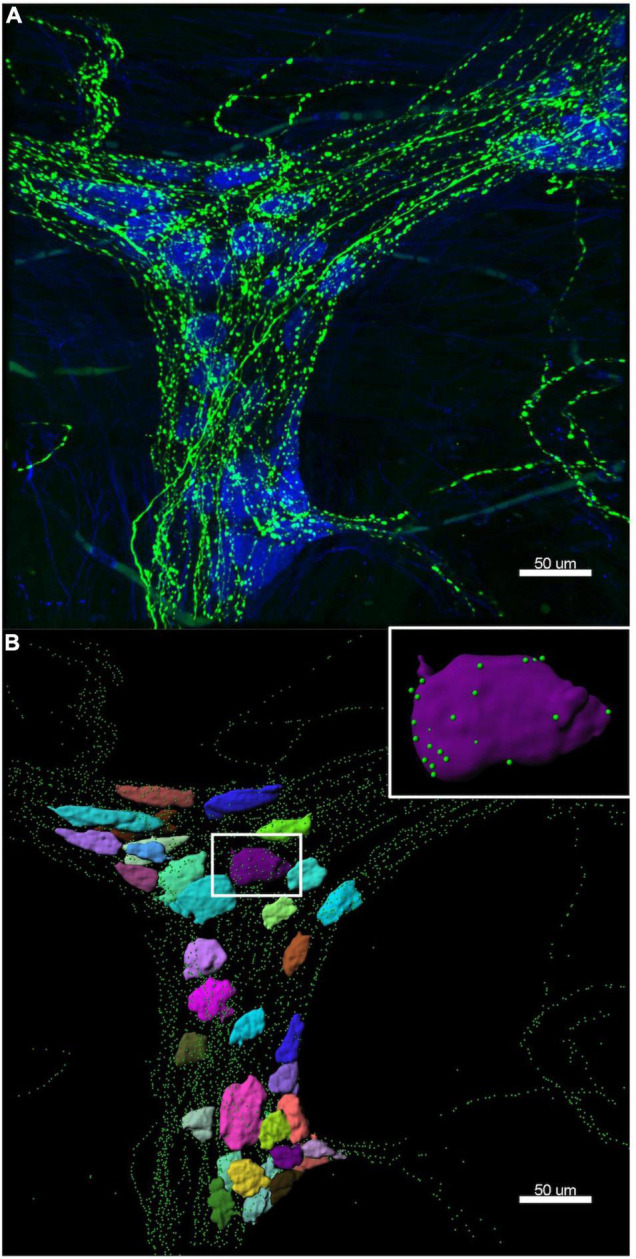
Example of a 3D reconstruction. **(A)** Confocal image of the same ganglion shown in Figure 1, with HuC/D cell bodies in blue and tyrosine hydroxylase (TH) varicosities in green. **(B)** The Imaris 3D reconstruction of HuC/D cell bodies is shown as multi-coloured surfaces to demonstrate individual cells, and TH varicosities are rendered as green spots. The colours assigned to the cells have no significance. Inset is a single HuC/D reconstructed cell body, with only the TH varicosities within 1 μm of the cell surface shown. Imaris allows accurate measurement of distances between varicosities and cell body surfaces. Data extracted from these 3D reconstructions was used to calculate the density of TH varicosities close to each myenteric cell body.

HuC/D cell bodies were reconstructed using the “surface” function in Imaris. Each individual cell body was rendered as a surface, then numbered in a micrograph of the ganglion. TH and ENK varicosities were reconstructed using the “spots” function, where each individual varicosity was represented as a single point. Parameters for rendering cell body surfaces and varicosity spots were kept uniform for the two ganglia of each preparation, but were changed between preparations due to variations in staining intensity. Figure 2 shows an example of a 3D reconstruction of a myenteric ganglion.

For each numbered cell “surface”, data was exported for its position (in coordinates x, y, and z), 3D shape, surface area (μm^2^) and volume (μm^3^), while the coordinates for each varicosity “spot” were also collected. At this stage, visually defined “baskets” of varicosities surrounding the numbered cell bodies were identified using the raw confocal images.

### Identification of Cell Type

Next, each numbered cell was classified, based on ChAT and NOS immunoreactivity in the second layer of immunohistochemical staining (ChAT +/NOS−, ChAT−/NOS +, ChAT +/NOS + or ChAT−/NOS−). Cell “surfaces” were excluded if they were incomplete (e.g., on the edge of the image), or if a single “surface” represented two or more apposed cells which could not be visually separated. It was not possible to classify cells on the basis of enkephalin immunoreactivity (i.e., ENK +/ENK−) because this neuropeptide is not reliably detected in nerve cell bodies without pre-incubation in colchicine (Furness et al., 1983; Brehmer et al., 2005).

### Varicosity Density Analysis

A novel analysis algorithm was developed by one of the authors (LW) to measure the density of varicosities close to cell surfaces, using data exported from Imaris. Density measurement requires calculating the number of varicosities within a given space and the volume of that space (μm^3^). It is independent of the size of the targetted neuron. The algorithm was programmed to determine the outlines of a 2 μm “shell,” incorporating the 3D space from 1 μm inside the cell surface to 1 μm outside the cell surface. Varicosities within this “shell” were counted and divided by the volume of the shell to derive an absolute density. The distance of 1 μm either side of the rendered cell body “surface” was chosen to measure nearby varicosities whose transmitter release is likely to have the largest effect on the cell or its synaptic inputs from other enteric neurons. The inclusion of varicosities up to 1 μm inside the cell accounted for varicosities located within clefts and allowed a margin of error of the rendered cell surface (cell “surface” detail was set to 1 μm). Varicosities were attributed only to the one cell they were closest to, in order to avoid double-counting. This process was carried out for both TH (sympathetic) and ENK (enteric) varicosities.

### Statistical Analysis of Varicosity Density

Only ChAT +/NOS−, ChAT−/NOS +, and ChAT +/NOS + cells were analysed and compared. ChAT−/NOS− were excluded as there were too few of these cells for meaningful analysis (Murphy et al., 2007).

To compare the densities of varicosities between the three remaining cell types (ChAT +/NOS−, ChAT−/NOS +, and ChAT +/NOS +), a generalised linear mixed-effects model of a negative-binomial family with log-link was used. A negative-binomial distribution model better accounts for the potential overdispersion of varicosities within the 2 μm thick shell, compared to a Poisson model. The 2 μm-thick shell volume was used as a multiplicative offset for each cell, which allows the regression coefficients to be effectively converted to varicosity densities. The brms R package was used to fit the model (Bürkner, 2017). The formulae below were used:

*Count*∼*cell_type* × *varic_type* + *(cell_type* × *varic_type | subj/gang)* + *offset[log(volume)]*
*Shape* ∼ *cell_type* × *varic_type*

where Count is an integer variable for the number of varicosities in a cell; cell_type is a categorical variable encoding the cell as one of the three combinations (ChAT +/NOS−, ChAT−/NOS +, or ChAT + /NOS +); varic_type is a categorical variable encoding varicosities for the count as either Enkephalin or TH; volume is a continuous offset equal to the volume of the 2 μm-thick shell surrounding the cell over which the count was taken, which induces units of density in the coefficients for cell_type and varic_type. The random-effect/multi-level variables subj and gang indicate the subject from which the tissue was obtained and the ganglion in which the cell was imaged, so that cells in the same subject and ganglion can share potential random variations in varicosity density. A prior of normal (0, 1) for coefficients, and LKJ(2) for correlations was used. Four chains were sampled, retaining 1,000 samples per chain, following 1,000 warmup samples. Diagnostics did not show any divergences and showed an Rhat close to 1.

To then compare the relative distributions of varicosity density close to cell types, a density ratio was calculated with 95% credible intervals (density of varicosities close to one cell type divided by the density of varicosities close to a different cell type). This method of statistical analysis was used to compare varicosity densities between ChAT +/NOS−, ChAT−/NOS +, and ChAT +/NOS + cells in three combinations:

(i) ChAT +/NOS− vs. ChAT−/NOS +
(ii) ChAT +/NOS− vs. ChAT +/NOS +
(iii) ChAT−/NOS + vs. ChAT +/NOS +

### Statistical Analysis of Cell Surface Area and Volume

Cell body surface area and volume were determined by Imaris and then presented as a mean ± standard error of the mean (SEM) for each cell type. A one-way analysis of variance (ANOVA) statistical test followed by Tukey’s *post-hoc* test was used to determine a difference in mean surface area and volume between cell types. Statistical analysis was performed using Prism version 8.4.1 for Mac (GraphPad Software, La Jolla, CA, United States). Statistical values <0.05 were considered statistically significant.

### Tyrosine Hydroxylase Immunoreactive Nerve Cell Bodies

Tyrosine hydroxylase-immunoreactive cell bodies from all ganglia in all seven preparations were manually counted using the raw confocal images. These were presented as a proportion of the total number of HuC/D cells counted in the imaged ganglia.

## Results

A total of 673 HuC/D-immunoreactive cell bodies were reconstructed from 14 ganglia from the seven patients. One hundred eighty-seven reconstructed cells were excluded because they were incomplete (cut-off by the edges of the imaged area), or unable to be separated from neighbouring cells. This left 486 cells for density analysis. As mentioned above, ChAT−/NOS− cell types (33 cells) were excluded from the varicosity density analysis due to their small numbers (Murphy et al., 2007).

### Tyrosine Hydroxylase Varicosity Density

**FIGURE 3:**
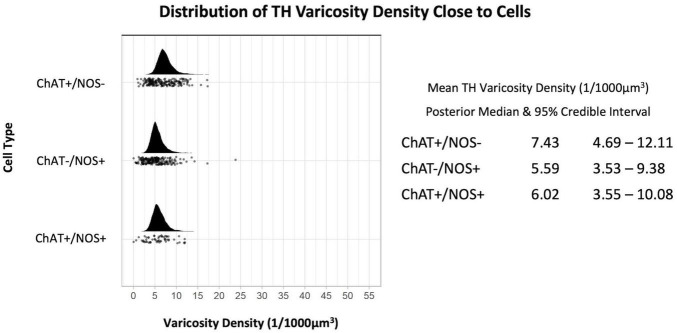
Graph of the distribution of tyrosine hydroxylase varicosities close to cells. The upright curve in each graph is the distribution of the mean density for each cell type. Every small black dot beneath represents the density of TH varicosities close to an individual cell. The posterior median of the mean varicosity density is greatest for the ChAT +/NOS– cell type at 7.43 varicosities per 1,000 μm^3^. The posterior median of the mean TH varicosity density for each cell type is presented with the 95% credible intervals on the right. ChAT, choline acetyltransferase; NOS, nitric oxide synthase; TH, tyrosine hydroxylase.

The TH varicosity density was greatest close to ChAT +/NOS− cells with a median (of the mean distribution) of 7.43 varicosities per 1,000 μm^3^ (190 cells), measured within ± 1 μm of the cell surface. This was followed by ChAT +/NOS + cells (6.02 TH varicosities per 1,000 μm^3^, 58 cells) then ChAT−/NOS + cells (5.59 varicosities per 1,000 μm^3^, 205 cells). The posterior median of the mean TH varicosity density and distribution of TH varicosities close to cells is shown in Figure 3.

**FIGURE 4:**
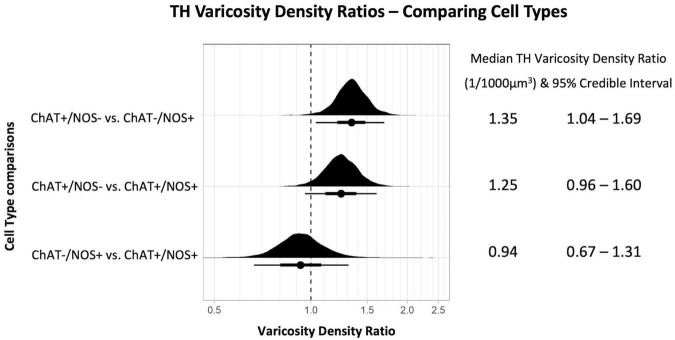
Graph of tyrosine hydroxylase varicosity density ratios comparing cell types. Density ratio is plotted on a logarithmic scale. The filled black curves indicate the ratio between the distributions of the mean of density (mean varicosity density of cell type divided by another). The bars beneath indicate the median (dot), the 66th percentiles and the 95th percentiles of the mean. A ratio of 1.0 indicates that the densities for the two types of cells compared are identical. In this graph, ChAT +/NOS– cells compared to the ChAT–/NOS + cells have a median density ratio of 1.35 and the 95% percentile bar is to the right of 1.0. This shows there is a significantly greater varicosity density close to ChAT +/NOS– cells compared to ChAT–/NOS + cells. The median TH varicosity density ratios are presented with the 95% credible intervals on the right. ChAT, choline acetyltransferase; NOS, nitric oxide synthase; TH, tyrosine hydroxylase.

Mean densities of TH-immunoreactive varicosities close to cell types differentiated by ChAT and NOS immunoreactivities were compared. There was a significantly greater density of TH varicosities close to ChAT +/NOS− compared with ChAT−/NOS + cells, with a median density ratio of 1.35 (95% credible interval 1.04–1.69, see Figure 4). There was a trend toward a greater density of TH varicosities close to ChAT +/NOS− cells vs. ChAT +/NOS + cells.

### Enkephalin-Immunoreactive Varicosities

**FIGURE 5:**
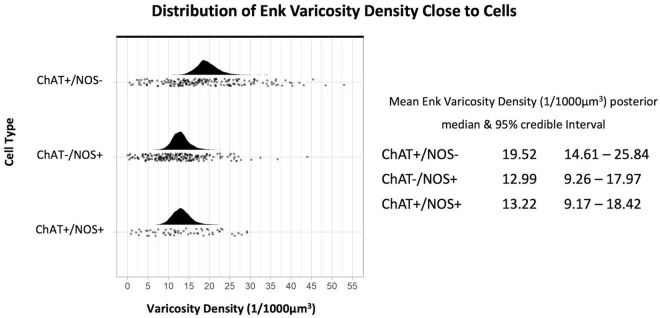
Graph of the distribution of enkephalin varicosities close to cells. The upright curve in each graph is the distribution of the mean density for each cell type. Each small black dot beneath represents the density of ENK varicosities close to an individual cell. The posterior median of the mean varicosity density is greatest for the ChAT +/NOS– cell type, at 19.52 varicosities per 1,000 μm^3^. The posterior median of the mean ENK varicosity density for each cell type is presented with the 95% credible intervals on the right. ChAT, choline acetyltransferase; NOS, nitric oxide synthase; ENK, enkephalin.

The density of ENK-immunoreactive varicosities around myenteric nerve cell bodies was calculated using the same method. Overall, ENK varicosities had a higher density than TH + varicosities. However, a notable feature of ENK varicosities is that they formed dense baskets around about 10% of myenteric nerve cell bodies; such baskets were very rare for TH varicosities. Disregarding the presence of baskets, the density of ENK varicosities was also greatest close to ChAT +/NOS− cells, with a posterior median of the mean distribution of 19.52 varicosities per 1,000 μm^3^, with lower densities for ChAT−/NOS + cells (12.99 varicosities per 1,000 μm^3^) and ChAT +/NOS + cells (13.22 varicosities per 1,000 μm^3^), see Figure 5.

**FIGURE 6:**
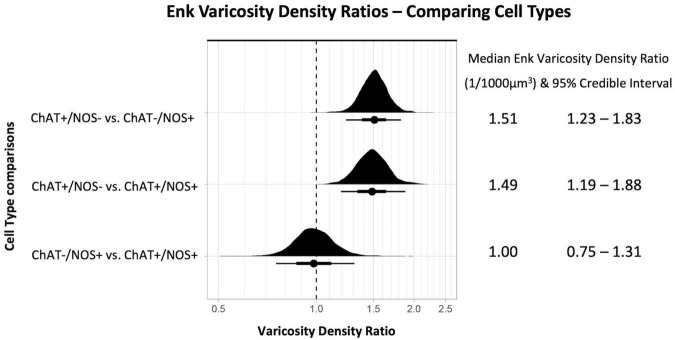
Graph of enkephalin (ENK) varicosity density ratios comparing cell types. Density ratio is on a logarithmic scale. The filled black curves indicate the ratio between the distributions of the mean of density (mean varicosity density of cell type divided by another). The bars beneath indicate the median (dot), the 66th percentiles and the 95th percentiles of the mean. A ratio of 1.0 indicates that the densities for the two types of cells compared are identical. In this graph, ChAT +/NOS– cells compared to the ChAT–/NOS + cells and also compared to the ChAT +/NOS + cells have a median density ratio and 95% percentile bar to the right of 1.0. This shows there is a significantly greater density of ENK varicosities close to ChAT +/NOS– cells compared to both ChAT–/NOS + and ChAT +/NOS + cells. The median ENK varicosity density ratios are presented with the 95% credible intervals on the right. ChAT, choline acetyltransferase; NOS, nitric oxide synthase; ENK, enkephalin.

Comparing the different myenteric neuron types, the density of ENK varicosities close to ChAT +/NOS− was greater than vs. ChAT−/NOS + cells, with a median density ratio of 1.51 (95% credible interval 1.23–1.83, see Figure 6). The same is true of ChAT +/NOS− vs. ChAT +/NOS + cells, which had a median density ratio of 1.49 (95% credible interval 1.19–1.88). There were no differences between the ChAT−/NOS + cells and the ChAT +/NOS + cells.

### Visually Defined Baskets

**FIGURE 7:**
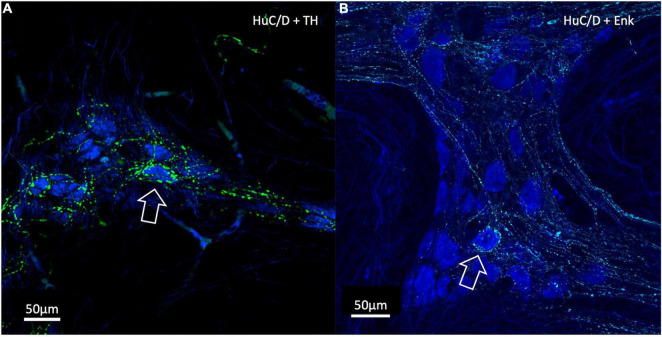
Confocal Images of HuC/D cells with surrounding varicosity baskets. TH varicosities are in green, ENK varicosities are in cyan and HuC/D myenteric cell bodies are in blue. **(A)** A TH-immunoreactive basket of varicosities surrounding an individual HuC/D cell (open arrow). **(B)** An ENK varicosity basket surrounding an individual HuC/D cell (open arrow). In both cases, these myenteric cells were ChAT +. Baskets represent a high density of varicosities with close appositions encircling a myenteric cell body and are likely to indicate targetted neural connectivity. ChAT, Choline Acetyltransferase; NOS, Nitric Oxide Synthase; TH, Tyrosine Hydroxylase; ENK, Enkephalin.

Of the nerve cells used for density analysis, 10 TH-immunoreactive “baskets” of varicosities were identified and these all surrounded HuC/D-immunoreactive nerve cell bodies (10/486 cells, 2.10%). Of these 10 baskets, all 10 surrounded myenteric neurons which were subsequently shown to be ChAT +/NOS−. There were 61 cells in the 7 specimens that were surrounded by ENK baskets (61/486, 12.6%), of which 53 were ChAT +/NOS− (87%); a highly significant association (χ^2^ = 75.0, *df* = 3, *p* < 0.01). There were no ChAT−/NOS + cells surrounded by ENK + baskets (see Table 2). Examples of HuC/D cells with TH and ENK baskets surrounding them are shown in Figure 7.

**TABLE 2:** Visually defined TH and ENK baskets surrounding myenteric plexus human RNA binding proteins C and D (HuC/D) cells.

	ChAT +/NOS−	ChAT−/NOS +	ChAT +/NOS +	ChAT−/NOS−	Total
TH Baskets	10 (100%)	0 (0%)	0 (0%)	0 (0%)	10
ENK Baskets	53 (87%)	0 (0%)	5 (8%)	3 (5%)	61

*TH, tyrosine hydroxylase; ENK, Enkephalin; ChAT, choline acetyltransferase; NOS, nitric oxide synthase.*

### Human RNA Binding Proteins C and D (HuC/D) Cell Characteristics

From the 486 reconstructed myenteric cell bodies, 190 (38%) were ChAT +/NOS−, 205 (42%) were ChAT−/NOS +, 58 (12%) were ChAT +/NOS +, and 33 (8%) were ChAT−/NOS−. HuC/D cell characteristics are presented in Table 3.

**TABLE 3:** HuC/D myenteric cell body proportions, surface area and volume.

Cell type	Number and percentage (mean% ± SEM)	Surface area (μm^2^, mean ± SEM)	Volume (μm^3^, mean ± SEM)
ChAT +/NOS−	190 38% ± 2.0	3,127 ± 428	4,566 ± 404
ChAT−/NOS +	205 42% ± 2.3	2,299 ± 371	3,177 ± 389
ChAT +/NOS +	58 12% ± 2.1	3,871 ± 494	6,078 ± 391
ChAT−/NOS−	33 8% ± 1.7	1,610 ± 238	2,156 ± 265

*SEM, standard error of the mean; ChAT, choline acetyltransferase; NOS, nitric oxide synthase.*

ChAT +/NOS + cells had the largest surface area and the largest mean cell volume, followed by ChAT +/NOS−, ChAT−/NOS +, and ChAT−/NOS− cells. There was a statistically significant difference in surface area between cell types using a one-way ANOVA test [*F*_(3,8)_ = 24.61, *p* < 0.0001), and using Tukey’s *post-hoc* test, ChAT +/NOS + cells were larger than ChAT−/NOS− cells (mean difference 2,261 μm^2^, 95% CI 1,243–3,279, *p* = 0.001) and ChAT−/NOS + cells (mean difference 1,572 μm^2^, 95% CI 833–2,311, *p* = 0.0013). ChAT +/NOS− cells were larger than ChAT−/NOS− cells (mean difference 1,517 μm^2^, 95% CI 618–2,415, *p* = 0.0045).

There was also a statistically significant difference in cell volumes [one-way ANOVA test, *F*_(3,18)_ = 47.09, *p* < 0.0001]. ChAT +/NOS + cells had a larger volume than all other cell types using *post-hoc* analysis: vs. ChAT−/NOS− (mean difference 3,922 μm^3^, 95% CI 2,841–5,002, *p* < 0.0001); vs. ChAT−/NOS + (mean difference 2,900 μm^3^, 95% CI 2,097–3,704, *p* < 0.0001); vs. ChAT +/NOS− (mean difference 1,512 μm^3^, 95% CI 174–2,850, *p* = 0.03). ChAT +/NOS− cells were larger than ChAT−/NOS− cells (mean difference 2,409 μm^3^, 95% CI 1,308–3,511, *p* < 0.0011).

### Intrinsic Tyrosine Hydroxylase-Immunoreactive Myenteric Nerve Cell Bodies

**FIGURE 8:**
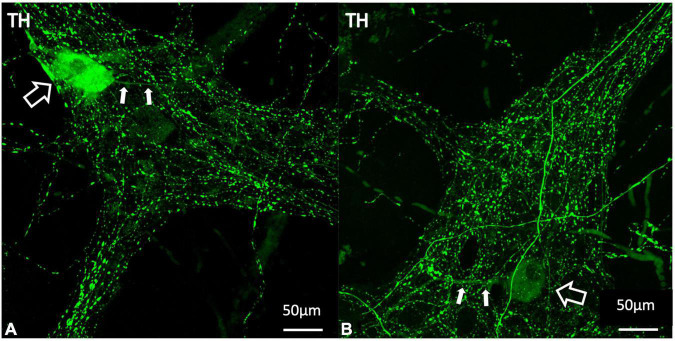
Examples of TH positive myenteric cell bodies **(A,B)**. TH labelling is shown in green. The larger open white arrows show TH immunoreactive cell bodies and the smaller filled arrows demonstrate single long axonal projections from each of these cells, seen in 10 of the 22 identified TH positive cell bodies. TH immunoreactive cell bodies make up 1% of the total population of myenteric cells and are likely to indicate intrinsic catecholaminergic cells in the myenteric plexus, some of which may be dopaminergic in nature. TH, tyrosine hydroxylase.

Forty-four ganglia from seven patients were included in the analysis of intrinsic TH positive cell bodies. An example of a TH -immunoreactive cell body is shown in Figure 8. This analysis did not require 3D reconstruction. Of 2,252 HuC/D cell bodies in 44 ganglia, 22 (1%) were TH positive. After elution of TH and relabelling with NOS and ChAT antisera, 21 of these 22 cell bodies were ChAT +/NOS−, while one was ChAT−/NOS−. None contained NOS immunoreactivity.

## Discussion

The sympathetic nervous system has a net inhibitory effect on intestinal motility. Sympathetic axons release noradrenaline, which binds to alpha 2a adrenergic receptors to cause presynaptic inhibition of the release of acetylcholine, thus inhibiting interneuronal pathways and reducing smooth muscle excitation and contraction (Nishi and North, 1973; Hirst and McKirdy, 1974). Theoretically, if noradrenaline also inhibited excitatory cholinergic synaptic drive to inhibitory motor neurons, sympathetic activation could lead to net excitation. Using 3D reconstruction and novel density analysis, our study demonstrated that noradrenergic varicosities have a significantly greater density close to ChAT +/NOS− cells compared to ChAT−/NOS + cells in the myenteric plexus. This arrangement suggests that extrinsic post-ganglionic sympathetic inputs to the myenteric plexus act via cholinergic cell bodies in human colonic myenteric ganglia. It should be noted that myenteric nerve cell bodies may not be the primary target of sympathetic axons. Rather, the adjacent cholinergic synaptic terminals are likely to be a major site of action via presynaptic inhibition (Hirst and McKirdy, 1974). However, enteric neurons generally have very restricted dendritic fields (Dogiel, 1899), so measuring the distance of varicosities to HuC/D immunoreactive nerve cell bodies is likely to be a reasonable approximation of proximity to presynaptic terminals.

Enkephalin varicosities similarly had a greater density close to ChAT +/NOS− cell types compared to nitrergic neurons. Previous studies have shown that ENK-immunoreactive interneurons, which give rise to varicose endings in myenteric ganglia, belong to several populations of cholinergic ascending interneurons (Brookes et al., 2020; Humenick et al., 2020). Baskets indicate “targetted wiring” where there is a concentration of close contacts between varicosities and neuron cell bodies. In our present study, most visually defined ENK baskets surrounded ChAT +/NOS− cells. TH varicosity baskets are much less abundant but also targetted specific cells; all 10 nerve cell bodies surrounded by TH baskets were ChAT +/NOS−. The 3D reconstruction and density analysis provides a quantifiable method of demonstrating targetted connectivity onto enteric neurons for both identifiable baskets and the distribution of varicosities that were not part of baskets.

Relative proportions of cell types defined by ChAT and NOS immunoreactivities in our study are similar to those reported previously in human colon (Murphy et al., 2007; Wattchow et al., 2008; Beck et al., 2009; Ng et al., 2018). The four cell types differed in both cell body surface area and volume. This suggests they are distinct and may form different functional types. HuC/D labelling is a pan-neuronal marker in the ENS, and the vast majority HuC/D cells are immunoreactive for either ChAT and/or NOS (Murphy et al., 2007). ChAT +/NOS− cells are likely to represent several different functional types of neurons in the myenteric plexus, including excitatory motor neurons to the longitudinal and circular muscle, ascending interneurons, some descending interneurons, primary afferent neurons, some vasomotor neurons and some secretomotor neurons (Porter et al., 2002; Murphy et al., 2007). Based on the results of our study, noradrenergic inputs innervate one or several of these functional cell types in a targetted manner.

Electron microscopy is considered the gold standard in identifying synaptic connections morphologically. Previous research using both electron microscopy and light microscopy has shown that varicosities arranged in dense pericellular baskets often form synaptic connections onto the encircled cell bodies (Pompolo and Furness, 1995). In a study by Mann et al. (1997) numerous direct appositions between varicosities and neurons identified by confocal microscopy, corresponded to numerous synaptic connections confirmed by electron microscopy. In our study, reconstructed varicosities at a distance of 1 μm from the reconstructed HuC/D cell body surface (within or outside the reconstructed surface) were counted as close appositions. Therefore, it is possible that the targetted innervation of ChAT +/NOS− neurons by noradrenergic varicosities may be synaptic in nature, however, this cannot be definitively concluded from our study without directly observing ultrastructural features of synapses by electron microscopy. Llewellyn-Smith et al. (1984) previously demonstrated ultrastructural synapses using electron microscopy between noradrenergic neurons and unspecified myenteric neurons in human small intestine, and several animal studies have also identified noradrenergic synaptic connections in the myenteric plexus of the GIT (Manber and Gershon, 1979; Gershon and Sherman, 1982, 1987; Hayakawa et al., 2008). Additionally, volume transmission *via* the diffuse release of noradrenaline within a ganglion to act on distant cells which express appropriate receptors, cannot be ruled out (Vanhatalo and Soinila, 1998).

The novel method in our study, including 3D reconstruction and density analysis has several benefits. Reconstruction of TH-immunoreactive varicosities and myenteric cell bodies from confocal images enabled precise measurement of distances between the two objects in 3 dimensions. Importantly, the reconstruction of varicosities and myenteric cell bodies was done only with reference to the first layer of immunohistochemistry, i.e., TH, ENK, and HuC/D immunoreactivity. Thus the investigator was blinded to the cell type defined by ChAT and NOS antisera, which were applied in the second layer. Density of varicosities in a shell volume within ± 1 μm of the cell surface was chosen as this value is independent of the size of cells. The surface areas and volumes of ChAT and NOS–immunoreactive neurons differed; close packing within the ganglia would predict larger absolute numbers of varicosities close to larger cells; density measurement avoids this.

It should be noted that our study contained specimens from only seven patients. All ganglia studied were from the myenteric plexus between the taenia. It has been shown that the plexus is denser under the taenia than between taenia and we cannot exclude that there may be differences at the cellular level too (Hanani et al., 2012). During the 3D-reconstruction process, many reconstructed cells had to be removed because the rendering was imperfect, or because they lay partially outside the boundaries of the image. Some cells had to be cut to separate them from a closely apposed cell. In addition, the images had lower resolution in the *z*-axis than in *x* and *y*-axes. In theory this could increase errors for points located above cell surfaces, although it appears unlikely that this could explain the differences between NOS + and ChAT + cells. The tissue was all maximally stretched in both axes prior to fixation and it is possible that this affected the absolute distances of varicosities from cell surfaces due to distortion of the ganglia (Gabella and Trigg, 1984). In addition, distances were measured at the moment of fixation; we cannot rule out the possibility that varicosities may be capable of some movement over time. In addition, we have assumed that a higher density of varicosities close to a nerve cell body will, on average, have a larger effect than a lower density of varicosities. No attempt was made to quantify this or to assess the tortuosity of diffusion pathways within myenteric ganglia. Lastly, it is possible that some of the TH varicosities may arise from the rare intrinsic TH-immunoreactive myenteric nerve cell bodies. However, only 1% of myenteric neurons were found to be TH positive, therefore it is likely that intrinsic catecholaminergic varicosities made up a very minor contribution to the total number of TH varicosities. Some intrinsic TH-immunoreactive cell bodies are likely to be dopaminergic and may play a role in modulating gastrointestinal motility (Wakabayashi et al., 1989; Anlauf et al., 2003). In our study we found these intrinsic catecholaminergic cells almost exclusively belonged to the ChAT +/NOS− type.

In this research we have verified a novel method for quantifying the density of innervation of neurons by different types of varicose nerve terminals. Our findings suggest that post-ganglionic sympathetic (noradrenergic) inputs preferentially target a subset of cholinergic neurons in the human colon myenteric plexus, and are likely to cause presynaptic inhibition to those cells involved in excitatory pathways. The connectivity demonstrated here may represent an important pathway in the modulation of colonic motility by the sympathetic nervous system.

## Data Availability Statement

The datasets presented in this study can be found in online repositories. The names of the repository/repositories and accession number(s) can be found below: Pennsieve, University of Pennsylvania: SPARC Consortium, Accession Number 1509, DOI: 10.26275/aqri-vyb4.

## Ethics Statement

The studies involving human participants were reviewed and approved by the Southern Adelaide Clinical Human Research Ethics Committee, Approval number 207.17. The patients/participants provided their written informed consent to participate in this study.

## Author Contributions

DP conducted the research as the lead and wrote the manuscript. DP, SB, PD, and DW developed the study concept and design. LW developed the software and statistical methods to analysed data. AH and BC contributed to the laboratory experiments. DP, SB, PD, DW, LW, AH, BC, and TS reviewed the final manuscript and images. All authors contributed to the article and approved the submitted version.

## Conflict of Interest

The authors declare that the research was conducted in the absence of any commercial or financial relationships that could be construed as a potential conflict of interest.

## Publisher’s Note

All claims expressed in this article are solely those of the authors and do not necessarily represent those of their affiliated organizations, or those of the publisher, the editors and the reviewers. Any product that may be evaluated in this article, or claim that may be made by its manufacturer, is not guaranteed or endorsed by the publisher.
